# Diagnostic Accuracy of APTw and NOEw MRI Metrics for IDH Mutation and 1p/19q Codeletion Prediction in Gliomas

**DOI:** 10.1002/nbm.70372

**Published:** 2026-08-07

**Authors:** Capucine Cadin, Stefano Casagranda, François Xavier Lejeune, Marianne Golse, Bertrand Mathon, Ottavia Dipasquale, Christos Papageorgakis, Mauro Zucchelli, Emmanuel Mandonnet, Stéphane Lehéricy, Franck Bielle, Marc Sanson, Patrick Liebig, Moritz Zaiss, Lucia Nichelli, Francesca Branzoli

**Affiliations:** ^1^ Paris Brain Institute ‐ ICM, Inserm U 1127, CNRS UMR 7225, Sorbonne Université, UMR S 1127, Equipe labellisée LNCC Paris France; ^2^ Department of R&D Advanced Applications Olea Medical La Ciotat France; ^3^ Data Analysis Core Paris Brain Institute Paris France; ^4^ Department of Neuroradiology La Pitié Salpêtrière Hospital, AP‐HP Paris France; ^5^ Department of Neurosurgery La Pitié Salpêtrière Hospital, AP‐HP Sorbonne University Paris France; ^6^ Department of Neurosurgery Lariboisière Hospital, AP‐HP Paris France; ^7^ Université de Paris Cité Paris France; ^8^ Paris Brain Institute ‐ ICM, Center for NeuroImaging Research – CENIR, Inserm U 1127, CNRS UMR 7225, Sorbonne Université, UMR S 1127 Paris France; ^9^ Siemens Healthcare GmbH Erlangen Germany; ^10^ Department of Neuroradiology Friedrich‐Alexander Universität Erlangen‐Nürnberg (FAU) Erlangen Germany

**Keywords:** 1p/19q codeletion, APTw‐CEST, glioma diagnosis, IDH, NOEw, protein‐related markers

## Abstract

Isocitrate dehydrogenase (IDH) mutation and 1p/19q codeletion are key molecular markers for glioma classification. Amide proton transfer weighted (APTw) and nuclear Overhauser effect–weighted (NOEw) markers showed promise for glioma characterization, by probing protein‐related tissue properties. However, their interpretation is confounded by direct water saturation, macromolecules (semi‐solid magnetization transfer—ssMT), and *T*_1_ relaxation. Here, we aimed to assess the performance of three APTw and NOEw metrics—uncorrected, spillover/ssMT‐corrected (FMC), and fully spillover/ssMT‐ and *T*_1_‐corrected (FMTC)—for glioma stratification. Fifty patients with suspected gliomas were prospectively enrolled (12 IDH‐wild‐type, 38 IDH‐mutant, of which 21 with 1p/19q codeletion). Acquisitions were performed at 3 T using a 3D gradient echo readout with chemical exchange saturation transfer (*B*_1_ = 2 μT for APTw, 0.6 μT for NOEw; *T*_1sat_ = 2 s), WASABI (WAter Shift And B1) for *B*_0_/*B*_1_ mapping, and saturation recovery for *T*_1_ mapping. Glioma subtypes were compared using metrics extracted from manually segmented masks, using two‐tailed Mann–Whitney U tests, and the Benjamini–Hochberg false discovery rate (FDR) correction. Effect sizes were quantified using Cliff's *δ* with 95% bootstrap confidence intervals and classification performance was assessed by receiver operating characteristic analyses (area under the curve, AUC). IDH‐mutant and wild‐type gliomas differed significantly for the uncorrected APTw metric (*p* = 0.005, AUC = 0.79), with stronger discrimination following correction—APTw‐FMC (*p* < 0.001, AUC = 0.94) and APTw‐FMTC (*p* < 0.001, AUC = 0.96), both with large effect sizes. Only APTw‐FMTC distinguished 1p/19q codeleted from non‐codeleted gliomas before FDR correction (uncorrected *p* = 0.01, AUC = 0.74). The NOEw metrics did not differ between any molecular subgroups, likely due to limited sensitivity of this contrast at 3 T. These results suggest that correcting for fluid, ssMT, and *T*_1_ effects enhances the accuracy of APTw metrics, offering a more robust and biophysically grounded approach to noninvasive glioma diagnosis.

## Graphical abstract

We evaluated how confounding effects from fluid, macromolecules and *T*_1_ relaxation impact the performance of APTw and NOEw metrics for noninvasive glioma classification at 3 T. Corrected APTw metrics showed the highest discriminations of IDH‐mutant from wild‐type gliomas (AUC = 0.94–0.96), whereas NOEw metrics did not differ between groups. The fully‐corrected APTw‐FMTC was the best to separate 1p/19q‐codeleted from non‐codeleted gliomas, although not significantly. These findings suggest that integrating corrected APTw imaging into routine MRI protocols may improve noninvasive glioma stratification.

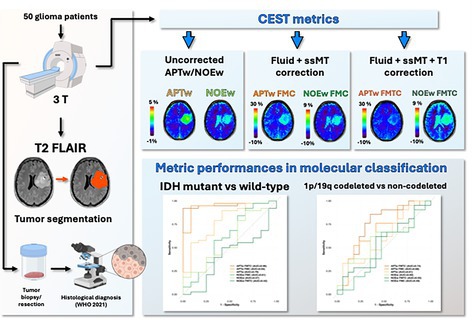

**Abbreviations**
APTw: amide proton transfer weighted
AREX: apparent exchange‐dependent relaxation
CEST: chemical exchange saturation transfer
DS: direct water saturation
FMC: fluid‐ and MT‐corrected
FMTC: fluid‐, MT‐ and *T*_1_‐corrected
IDH: isocitrate dehydrogenase
MT: magnetization transfer
NOEw: nuclear Overhauser effect–weighted
ssMT: semi‐solid magnetization transfer
WASABI: WAter Shift And B1

## 1 Introduction

Gliomas harboring mutations in the isocitrate dehydrogenase (IDH) gene constitute a biologically distinct subgroup of tumors with significant prognostic and therapeutic implications. IDH mutations are associated with a markedly favorable prognosis, and their noninvasive assessment plays a crucial role in diagnosis and treatment planning [1]. Among IDH‐mutant gliomas, the 1p/19q chromosomal codeletion status is needed to distinguish two major molecular subtypes: oligodendrogliomas and astrocytomas. Oligodendrogliomas are characterized by the simultaneous loss of chromosome arms 1p and 19q and are typically associated with a more favorable prognosis and greater responsiveness to treatment. In contrast, astrocytomas lack this codeletion and are associated with poorer patient outcomes [2]. Histopathological analysis combined with genetic testing is the gold standard for glioma classification, but it is invasive, time‐demanding, and subject to sampling bias due to tumor heterogeneity.

Chemical exchange saturation transfer (CEST) MRI has emerged as a powerful noninvasive imaging approach for probing tissue biochemistry and protein‐related composition [3, 4]. Among CEST‐derived contrasts, amide proton transfer weighted (APTw) imaging reflects the concentration of amide protons in mobile cellular proteins and peptides [4, 5], while nuclear Overhauser effect–weighted (NOEw) imaging provides complementary sensitivity to aliphatic proton interactions and protein folding [3, 6]. Unlike NOE [7], the APT contrast is also pH‐dependent, since the amide proton exchange rate is base‐catalyzed and slows down in acidic environments, leading to reduced signal intensity in more acidic tissues [5, 8].

Previous studies have shown that these MRI biomarkers can aid glioma grading and correlate with molecular profiles such as IDH status [9, 10]. However, the interpretation of APTw and NOEw signals is complicated by several non‐specific physical contributions. Firstly, the radiofrequency (RF) saturation pulse used to probe the amide (*Δω* = +3.5 ppm) or the NOE (*Δω* = −3.5 ppm) frequencies is not perfectly selective: its finite bandwidth and off‐resonance “tails” also partially saturate nearby proton pools, thereby diluting the specificity of the targeted signal. In addition, as the steady‐state signal depends on *T*_1_ relaxation, differences in *T*_1_ values influence the apparent saturation level.

As a result, three processes occur simultaneously: (i) partial excitation of the water protons leads to direct water saturation (DS, also known as “spillover” effect); (ii) the broad macromolecular pool undergoes semi‐solid magnetization transfer (ssMT), transferring saturation to water; and (iii) the steady‐state signal *T*_1_ relaxation dependence leads to *T*_1_ relaxation weighting. These effects, extensively discussed in [4, 11, 12], act as contaminating terms that obscure the true protein‐related CEST contrast, especially at clinical field strengths.

Because the ssMT and DS background are not perfectly symmetric around the relative water resonance (*Δω* = 0 ppm) [13], and because both effects act as broad, power‐dependent components that scale the entire Z‐spectrum rather than shifting it linearly, their contributions do not cancel out when computing the standard asymmetry metric $\mathrm{MT}R_{\text{asym}}=Z\left(-\Delta \omega \right)-Z\left(+\Delta \omega \right)$. Consequently, the measured APTw and NOEw contrasts contain both exchange‐ and non‐exchange‐related contributions. Spillover‐ and MT‐compensated metrics such as MTR_Rex_ correct for DS and ssMT contamination, and have been shown to suppress fluid contamination [14]. AREX, or apparent exchange‐dependent relaxation, further removes residual *T*_1_ dependence, yielding a contrast that more closely reflects the pure chemical exchange effect [12].

These confounding effects are particularly relevant in gliomas, where the presence of fluid‐rich tissue [14, 15], such as necrotic cavities, cystic components, or vasogenic edema, together with increased semi‐solid macromolecular content, can influence the apparent APTw and NOEw contrasts. However, to date most glioma CEST studies have relied on the uncorrected, asymmetry‐based MTR_asym_ metric, which conflates the true exchange signal with DS, ssMT, and *T*_1_ effects. Notably, these confounds are more pronounced at the clinical field strength of 3 T, whereas the most discriminative CEST findings to date have largely been obtained at 7 T [16, 17]. Moreover, although metrics such as MTR_Rex_ have recently been shown to improve glioma molecular characterization compared with conventional MTR_asym_ [18], a systemic, hierarchically corrected comparison that adds full *T*_1_ compensation (AREX) in a fluid‐suppression framework has not been performed so far.

To address these gaps, we performed a systemic, inter‐patient comparison of three hierarchically corrected CEST‐weighted MRI metrics: uncorrected MTR_asym_, spillover/ssMT‐corrected MTR_Rex_, and fully relaxation‐compensated AREX, applied to both APTw and NOEw acquisitions in a single prospective cohort at 3 T. To our knowledge, this is the first study to compare these three hierarchically corrected metrics—each progressively removing distinct sources of physical contamination—for both IDH and 1p/19q stratification at a clinical strength. Overall, we aimed to determine which CEST‐weighted biomarker best discriminates glioma molecular subtypes and to clarify the clinical utility of APTw and NOEw imaging as a noninvasive surrogate marker of intracellular protein content and glioma molecular status.

## 2 Methods

### 2.1 Participants

Fifty consecutive patients were prospectively included in this study, between March 2023 and February 2026. Inclusion criteria were as follows: (i) suspicion of a low‐grade glioma; (ii) planned surgery or biopsy; (iii) age > 18 years; (iv) ability and willingness to provide written informed consent. Tumor molecular subtyping (including IDH and 1p/19q codeletion status) was obtained from surgical samples according to the 2021 WHO classification. All procedures were approved by the local institutional review board, and the study was registered on the ClinicalTrial.gov database with the number NCT05700071. Written informed consent was provided by each participant prior to inclusion.

### 2.2 MRI Acquisition

All MRI scans were performed using a whole‐body 3 T scanner (MAGNETOM Prisma fit, Siemens Healthineers, Erlangen, Germany) equipped with a 64‐channel receive‐only head coil.

3D fluid attenuated inversion recovery (FLAIR) images (field‐of‐view [FOV] = 256 × 256 × 176 mm^3^, spatial resolution: 1.0 × 1.0 × 1.0 mm^3^, repetition time [TR]/echo time [TE] = 7000/393 ms), were acquired for planning the CEST acquisition and for tumor segmentation.

CEST‐weighted data were acquired using a 3D snapshot gradient‐echo (GRE) CEST sequence [19], with two saturation protocols:

- APT‐weighted acquisition: *B*_1_ = 2 μT, duty cycle = 90%, *T*_1sat_ = 2 s, optimized for amide proton transfer.
- NOE‐weighted acquisition: *B*_1_ = 0.6 μT, duty cycle = 50%, *T*_1sat_ = 2 s, optimized for aliphatic proton transfer.

Frequency offsets were applied from −6 to +6 ppm in 0.5‐ppm increments, and one unsaturated M_0_ volume was also acquired. The WASABI (WAter Shift And B1) sequence [15] was used for *B*_0_/*B*_1_ mapping. Quantitative *T*_1_ maps were obtained using a saturation recovery sequence (*B*_1_ = 5 μT, 14 recovery points). This approach acquires multiple saturation recovery images with variable delay times after a 5 μT saturation pulse, allowing voxel‐wise fitting of the longitudinal relaxation curve to estimate *T*_1_ values used in the AREX maps computation.

Sequence parameter setting includes: FOV = 219 × 180 mm^2^, matrix = 112 × 92, slice thickness = 5 mm, TR = 4 ms, TE = 2 ms, flip angle = 7°, bandwidth = 700 Hz/pixel.

### 2.3 Data Pre‐ and Post‐Processing

APTw and NOEw data were processed in Olea Sphere 3.0 (Olea Medical, La Ciotat, France) following the standardized pipeline described by Nichelli et al. [20] The workflow included: PCA‐based temporal denoising to suppress random noise while preserving spectral structure [21]; wavelet‐based spatial smoothing to enhance signal‐to‐noise ratio (SNR) without blurring fine details [22]; motion correction via intensity‐based registration using Elastix [23]; generation of the Z‐spectrum by normalizing each volume M(Δω) by *M*_0_ volume; *B*_0_ field correction using linear interpolation of the Z‐spectrum [13]; and *B*_1_ field correction using a decorrelation algorithm [24].

*B*_0_ and *B*_1_ maps used for these correction were computed from the WASABI data [25], while *T*_1_ maps were derived from saturation recovery data using a Levenberg–Marquardt nonlinear least‐squares algorithm.

### 2.4 CEST‐Derived Metrics

#### 2.4.1 MTR_asym_ Metric

The conventional magnetization transfer ratio asymmetry was calculated as follows:

$$\mathrm{MT}R_{\text{asym}}\left(3.5\mathrm{ppm}\right)=Z\left(-3.5\mathrm{ppm}\right)-Z\left(3.5\mathrm{ppm}\right)$$

This metric remains sensitive to DS, ssMT, and *T*_1_ relaxation effects.

In the present study, the MTR_asym_ maps were named according to the acquisition weighting:

- APTw (MTR_asym_ (3.5 ppm), *B*_1_ = 2 μT) for the amide proton transfer–weighted sequence, and
- NOEw (MTR_asym_ (3.5 ppm), *B*_1_ = 0.6 μT) for the nuclear Overhauser enhancement–weighted sequence.

#### 2.4.2 MTR_Rex_ Metric

The spillover‐ and ssMT‐corrected MTR was computed according to Zaiss et al. [12]:

$$\begin{array}{c} {\mathrm{MTR}}_{\mathrm{Rex}}\left(3.5\mathrm{ppm}\right)=\frac{1}{Z\left(3.5\mathrm{ppm}\right)}-\frac{1}{Z\left(-3.5\mathrm{ppm}\right)}\\ =\frac{Z\left(-3.5\mathrm{ppm}\right)-Z\left(3.5\mathrm{ppm}\right)}{Z\left(-3.5\mathrm{ppm}\right)\cdot Z\left(3.5\mathrm{ppm}\right)} \end{array}$$

This formulation removes the influence of DS and semi‐solid MT effects, providing a more specific exchange‐driven contrast.

The resulting maps were labeled as follows:

- APTw–FMC for the spillover‐ and ssMT‐corrected APT‐weighted acquisition, and
- NOEw–FMC for the spillover‐ and ssMT‐corrected NOE‐weighted acquisition.

#### 2.4.3 *AREX* Metric

To compensate for residual *T*_1_ relaxation bias, AREX [12] was computed as follows:

$$\text{AREX}=\frac{\mathrm{MT}R_{\mathrm{Rex}}}{T_{1}}$$

The resulting relaxation‐compensated maps were named:

- APTw–FMTC for the spillover‐, MT‐, and *T*_1_‐corrected APT‐weighted metric, and
- NOEw–FMTC for the spillover‐, MT‐, and *T*_1_‐corrected NOE‐weighted metric.

### 2.5 ROI Definition and Value Extraction

Tumor segmentation was performed on co‐registered FLAIR images by a certified neuroradiologist (L.N.—Reader no. 1) blinded to molecular status, using ITK‐SNAP software (Version 3.6) with a semi‐automatic active contour segmentation method and subsequent manual correction. For each subject, a volumetric region of interest (VOI) encompassing the entire hyperintense lesion on FLAIR was delineated slice‐by‐slice, carefully excluding large cystic or necrotic cavities when present. The contralateral normal‐appearing white matter (cNAWM) was segmented on the mirrored FLAIR image in the corresponding anatomic hemisphere, providing an internal normalization reference.

All VOIs were subsequently co‐registered to the CEST‐weighted maps (APTw and NOEw) to ensure spatial correspondence across modalities.

From each co‐registered VOI, the 90th percentile value of each CEST‐derived metric was extracted for statistical analysis.

This percentile‐based approach was chosen to capture the highest local CEST effects (“hotspot” regions) while reducing the influence of partial‐volume averaging and heterogeneous tissue components such as edema or necrosis.

In large and heterogeneous gliomas, mean or median values could tend to underestimate the true metabolic activity of the most biologically relevant subregions, whereas the 90th percentile has been shown to provide a more robust representation of the active tumor core [26].

Normalized contrasts were computed as follows:

$$\Delta {\mathrm{VOI}}_{\text{CEST}}={\mathrm{VOI}}_{\text{lesion}}-{\mathrm{VOI}}_{\text{cNAWM}}$$

and analyzed separately for APTw‐ and NOEw‐weighted datasets to assess their respective sensitivity to IDH mutation and 1p/19q codeletion status.

To assess inter‐reader reproducibility, a second neuroradiologist (M.G.—Reader no. 2), blinded to the first reader's segmentation and to molecular status, independently delineated the tumor VOI in all 50 patients following the identical protocol, and the CEST metrics were extracted from both readers' masks.

### 2.6 Statistics

All statistical analyses were performed using R software, Version 4.5.1.

Comparisons between IDH‐mutant versus wild‐type and 1p/19q codeleted versus non‐codeleted groups were conducted using the non‐parametric Wilcoxon–Mann–Whitney test. Effect sizes were quantified using Cliff's delta (*δ*), and median differences were assessed with 95% confidence intervals via bootstrap resampling (1000 iterations) to ensure robustness under unequal group sizes. Effect sizes were classified based on |*δ*| as negligible (≤ 0.147), small (0.147–0.33), medium (0.33–0.474), or large (≥ 0.474), following [27]. Receiver operating characteristic (ROC) analyses were conducted for all biomarkers, with performance summarized using the area under the curve (AUC).

For visualization, boxplots were generated for each metric and acquisition (APTw and NOEw), displaying the interquartile range and median values. To control for multiple comparisons, *p*‐values were adjusted using the Benjamini–Hochberg false discovery rate (FDR) correction. In selected cases, Welch's *t*‐tests by rank were performed for robustness assessment on median‐only comparisons. All tests were two‐tailed, and a corrected *p* < 0.05 was considered statistically significant.

Inter‐reader reproducibility of the extracted 90th‐percentile metrics was assessed with a two‐way random‐effect, single‐rater, absolute‐agreement intraclass correlation coefficient (ICC (1, 2)), computed in R with *irr* package, and interpreted following [28] (poor: < 0.50, moderate: 0.50–0.75, good: 0.75–0.90, excellent: > 0.90). Agreement was further examined by Bland–Altman analysis (mean bias and 95% limits of agreement).

## 3 Results

### 3.1 Participants

Histomolecular analysis revealed 12 IDH‐wild‐type gliomas (Grade 4) and 38 IDH‐mutant gliomas (25 of Grade 2, 10 of Grade 3, and 3 of Grade 4), of which 21 carried a 1p/19q codeletion. Demographic and histomolecular characteristics are reported in Table 1.

**TABLE 1:** Histomolecular and demographic characteristics of the study cohort. Characteristics of the 50 patients with diffuse glioma included in the study, grouped by IDH‐mutation status. Age is reported as mean (range); all other variables are patient counts. Dashes (–) denote categories not applicable to that group.

Tumor IDH status	# subjects	Age (years) mean (min–max)	Sex		Tumor grade			1p/19q codeletion	
			F	M	2	3	4	Codeleted	Non‐codeleted
Wild‐type	12	60.2 (46–87)	6	6	—	—	12	0	12
Mutant	38	41.8 (19–72)	13	25	25	10	3	21	17

Abbreviations: F, female; M, male.

### 3.2 Z‐Spectra Behavior and Effect of Corrections

**FIGURE 1:**
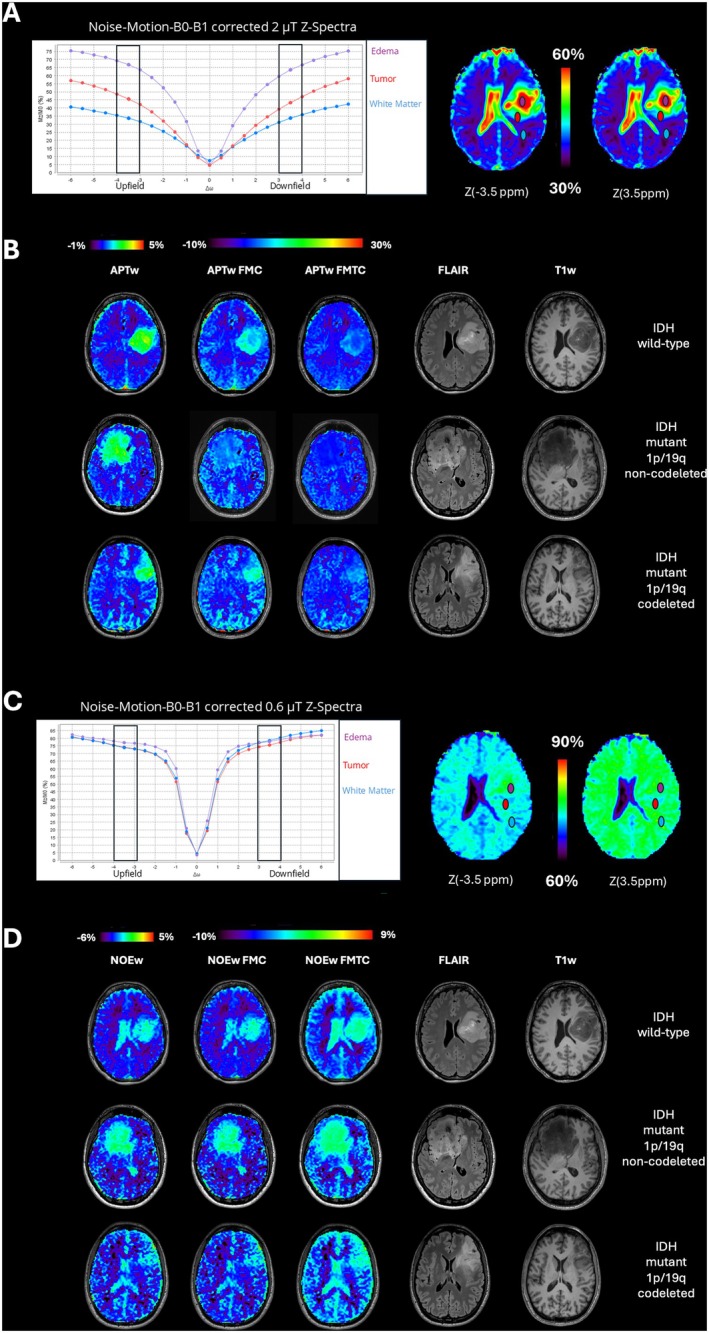
Representative examples of APTw and NOEw data in patients with gliomas. (A) Noise‐, motion‐, *B*_0_‐, and *B*_1_‐corrected Z‐spectra acquired at *B*_1_ = 2 μT (APTw acquisition) in an IDH‐wild‐type glioma, showing distinct signal behavior in tumor (red), peritumoral edema (purple), and contralateral normal‐appearing white matter (blue). Downfield offsets at +3.5 ppm correspond to the APT pool. (B) APTw (asymmetry‐based), APTw‐FMC (spillover‐ and MT‐corrected MTR_Rex_), and APTw‐FMTC (*T*_1_‐compensated AREX) maps derived for patients with IDH‐wild‐type, IDH‐mutant 1p/19q non‐codeleted, and IDH‐mutant 1p/19q codeleted gliomas, with corresponding FLAIR and *T*_1_‐weighted images. Progressive suppression of fluid‐related and ssMT contamination, yielded a contrast more specific to mobile protein amide protons. (C) Noise‐, motion‐, *B*_0_‐, and *B*_1_‐corrected Z‐spectra acquired at *B*_1_ = 0.6 μT (NOEw acquisition) for the same IDH‐wild‐type glioma. At lower saturation power, the upfield (−3.5 ppm) NOE component dominates the Z‐spectrum, emphasizing aliphatic and lipid proton transfer. (D) NOEw, NOEw‐FMC, and NOEw‐FMTC for the same patients with IDH‐wild‐type, IDH‐mutant 1p/19q non‐codeleted and IDH‐mutant 1p/19q codeleted gliomas, with corresponding FLAIR and *T*_1_‐weighted images. The gradual removal of fluid and semi‐solid MT contributions provides a contrast that predominantly reflects NOE‐related upfield interactions.

Figure 1A,C shows representative Z‐spectra and maps from a patient with an IDH‐wild‐type glioblastoma, acquired at 2 μT (APTw) and 0.6 μT (NOEw), respectively. At the higher saturation power, the APTw spectrum (+3.5 ppm) appeared broader, with stronger signal differences between tumor, edema, and NAWM, reflecting enhanced APT effects, but also greater ssMT and DS contamination. At the lower power (0.6 μT), the NOEw spectrum (−3.5 ppm) was narrower, with reduced background effects and lower overall contrast. Figure 1B,D shows APTw‐ and NOEw‐ maps alongside FLAIR anatomical images for three representative glioma subtypes (IDH‐wild‐type, IDH‐mutant non‐codeleted, and IDH‐mutant 1p/19q‐codeleted). Applying the hierarchical correction pipeline—from MTR_asym_ to MTR_Rex_ (FMC) and AREX (FMTC)—progressively reduced non‐specific contributions, an effect particularly pronounced in the APTw maps.

### 3.3 Inter‐Reader Reproducibility

Inter‐reader reproducibility of the 90th‐percentile metrics was excellent for all metrics (ICC (1, 2) 0.91–0.96), except NOEw‐FMC, which showed good agreement (ICC 0.90); values are reported in (Table S1). Bland–Altman analysis showed minimal systematic bias and limits of agreement that were narrow relative to each metric's value range (Figure S1). In the following sections tumor masks obtained by the Reader no. 1 were considered.

### 3.4 IDH‐Mutant Versus IDH‐Wild‐Type Gliomas

**FIGURE 2:**
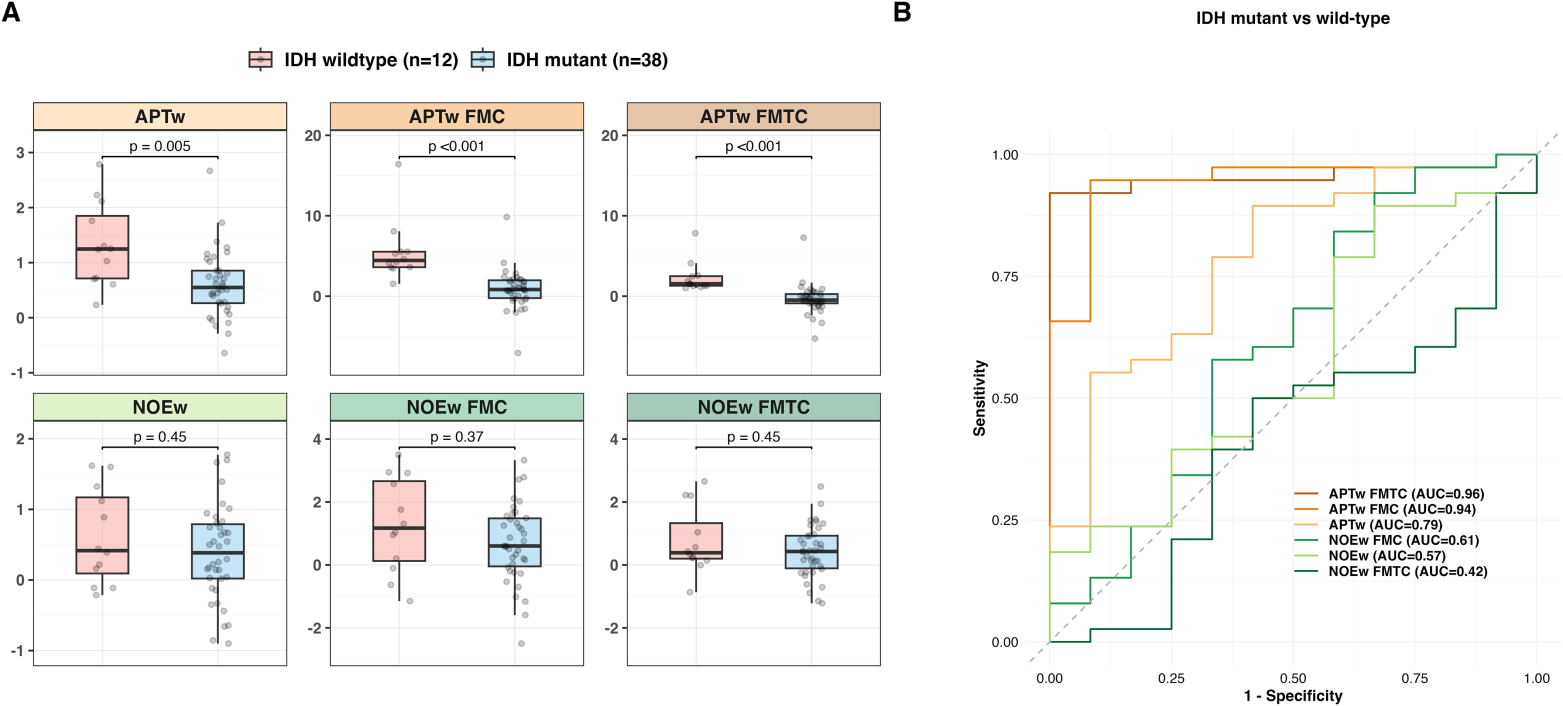
APTw and NOEw metrics in IDH‐mutant versus IDH‐wild‐type gliomas: boxplots and ROC analyses. (A) Distribution of the 90th‐percentile (p90) APTw‐ and NOEw‐derived metrics in IDH‐mutant versus IDH‐wild‐type gliomas. The three APTw‐based metrics (APTw, APTw–FMC, APTw–FMTC) were higher in IDH‐wild‐type than in IDH‐mutant tumors and differed significantly between groups, with the image‐corrected metrics showing the strongest separation (Mann–Whitney U; Benjamini–Hochberg FDR‐adjusted *p* < 0.01 for APTw and *p <* 0.001 for APTw–FMC and APTw–FMTC). None of the NOEw‐derived metrics (NOEw, NOEw–FMC, NOEw–FMTC) differed significantly. Boxes indicate the interquartile range (IQR), center lines the median, and whiskers extend to the most extreme values within 1.5 × IQR; jittered points represent individual subjects (group sizes in the legend). (B) ROC curves for discriminating IDH‐wild‐type from IDH‐mutant gliomas for each metric; AUCs are reported in the legend. FMC, spillover/ssMT‐corrected; FMTC, spillover/ssMT‐ and *T*_1_‐corrected. Complete statistics are reported in Table S2.

Figure 2 compares IDH‐mutant and IDH‐wild‐type gliomas using the 90th‐percentile value of each CEST‐derived metric, with the corresponding ROC curves and AUCs. All APTw‐derived metrics were higher in the IDH‐wild‐type group than the IDH‐mutant group. After FDR correction, the difference was statistically significant for uncorrected APTw (adjusted *p* = 0.005, AUC = 0.79), and stronger after image correction (APTw‐FMC, adjusted *p* < 0.001, AUC = 0.94; APTw‐FMTC, adjusted *p* < 0.001, AUC = 0.96). Effect sizes increased in parallel (|Cliff's *δ*| = 0.57, 0.89, and 0.91, respectively; all large). None of the NOEw‐derived metrics differed significantly between groups (all adjusted *p ≥* 0.37*,* AUC ≤ 0.61). Full statistics are provided in Table S2.

### 3.5 1p/19q Codeleted Versus Non‐Codeleted IDH‐Mutant Gliomas

**FIGURE 3:**
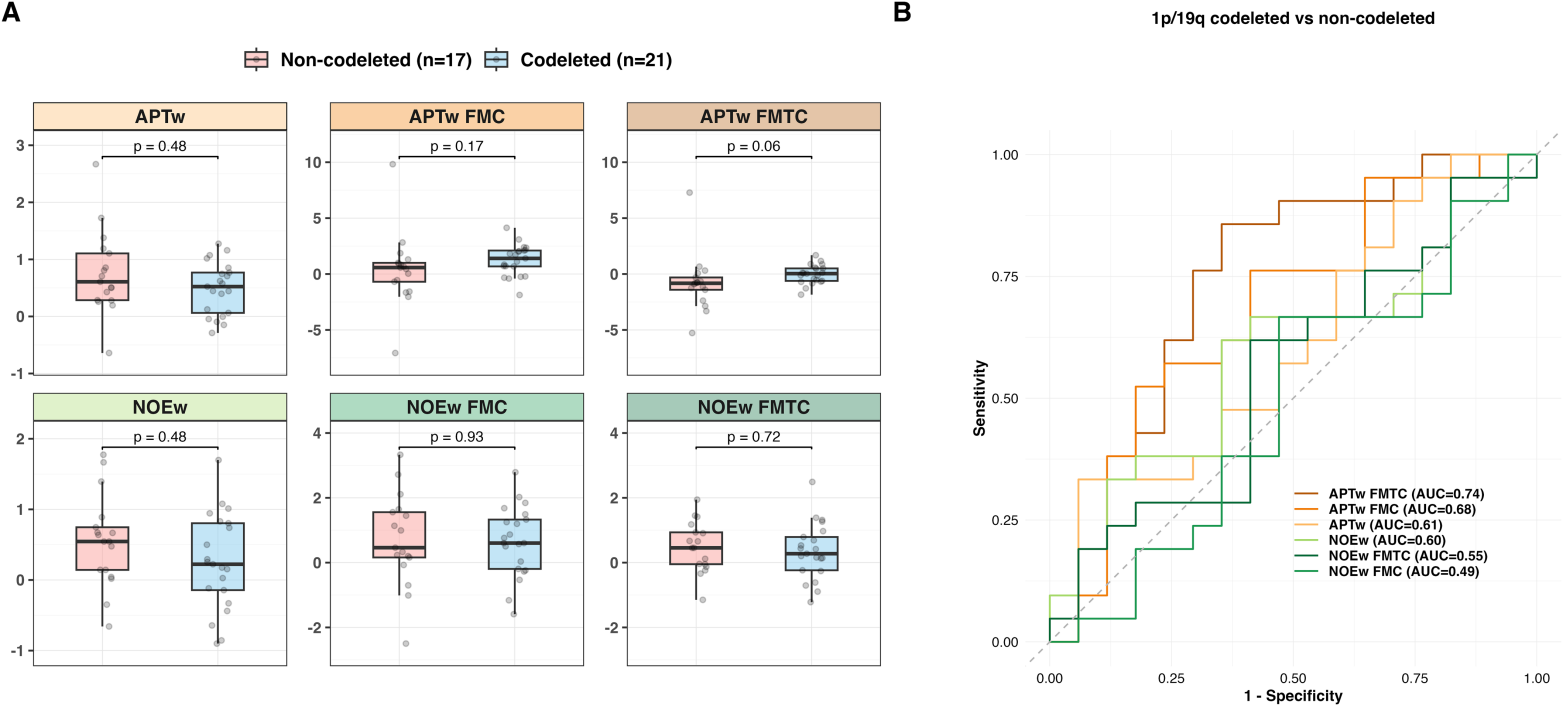
APTw and NOEw metrics in 1p/19q codeleted versus non‐codeleted IDH‐mutant gliomas: boxplots and ROC analyses. (A) Comparison of 1p/19q‐codeleted oligodendrogliomas and non‐codeleted astrocytomas within the IDH‐mutant group across the 90th‐percentile (p90) APTw‐ and NOEw‐derived metrics. APTw did not differ between codeleted and non‐codeleted tumors, and the image‐corrected APTw–FMC and APTw–FMTC were only numerically higher in codeleted gliomas. APTw‐FMTC differed at the uncorrected level (Mann–Whitney U, *p* = 0.01) but did not remain significant after Benjamini–Hochberg FDR correction (adjusted *p* = 0.06). None of the NOEw‐derived metrics differed significantly. Boxes, medians, and whiskers as in Figure 2; jittered points represent individual subjects. (B) ROC curves for discriminating codeleted from non‐codeleted tumors for each metric; AUCs are reported in the legend. Complete statistics are reported in Table S3.

Within the IDH‐mutant group, 1p/19q codeleted oligodendrogliomas were compared with 1p/19q‐non‐codeleted astrocytomas (Figure 3). On uncorrected APTw, signal was higher in astrocytomas, although the difference was not significant (adjusted *p* = 0.48*,* AUC = 0.61, |*δ*| = 0.21). Following metric correction, the direction of the differences reversed (oligodendrogliomas showed higher values than astrocytomas) and the separation increased progressively (APTw‐FMC, adjusted *p* = 0.17, AUC = 0.68; APTw‐FMTC, adjusted *p* = 0.06, AUC = 0.74; |*δ*| = 0.37 and 0.48, respectively). APTw‐FMTC reached significance only at the uncorrected level (*p* = 0.01) and did not survive FDR correction. NOEw‐derived metrics showed no significant differences between subgroups (all adjusted *p* > 0.77, |*δ*| < 0.28). Full statistics are provided in Table S3.

### 3.6 Comparison Across Histomolecular Subgroups

**FIGURE 4:**
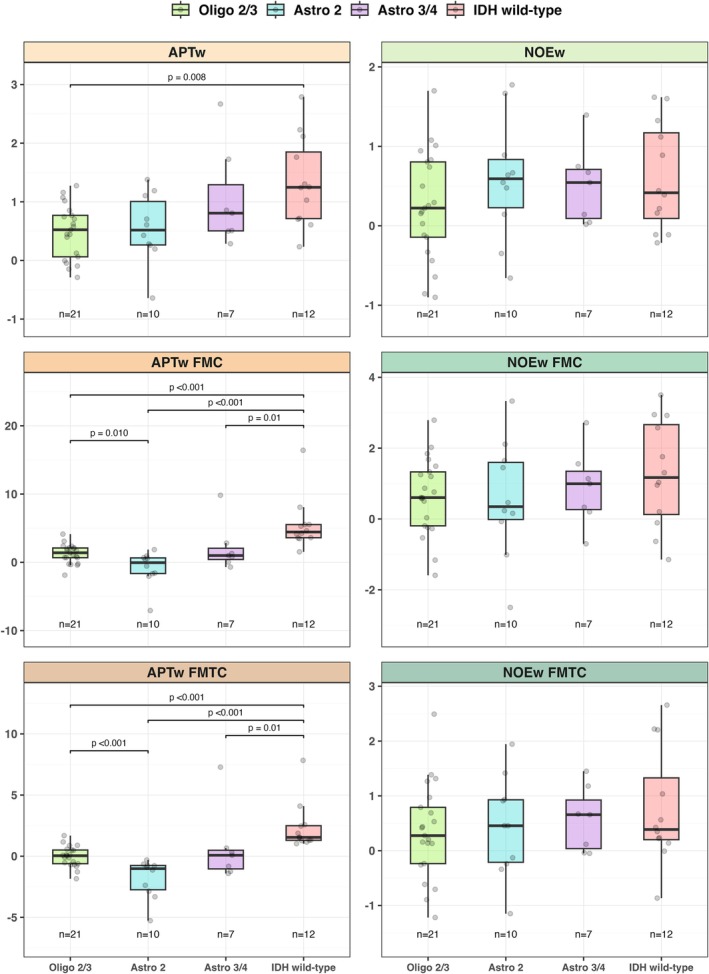
APTw and NOEw metrics across histomolecular glioma subgroups. Boxplots of the p90 APTw‐ and NOEw‐derived metrics across four histomolecular subgroups of increasing malignancy: 1p/19q‐codeleted oligodendrogliomas (Grades 2–3), IDH‐mutant non‐codeleted astrocytomas (Grade 2), IDH‐mutant non‐codeleted astrocytomas (Grades 3–4), and IDH‐wild‐type gliomas (glioblastoma, Grade 4). Uncorrected APTw increased progressively from lower‐ to higher‐subgroups, whereas the image‐corrected APTw‐FMC and APTw‐FMTC were comparable between oligodendrogliomas and high‐grade astrocytomas and significantly lower in Grade 2 astrocytomas than in oligodendrogliomas, high‐grade astrocytomas, and IDH‐wild‐types. NOEw‐derived metrics overlapped across all subgroups. Pairwise comparisons used Mann–Whitney U test with Benjamini–Hochberg FDR correction within each metric; only comparisons significant after correction (adjusted *p* < 0.05) are bracketed. Boxes indicate the interquartile range (IQR), center lines the median, and whiskers extend to the most extreme values within 1.5 × IQR; jittered points represent individual subjects, with per‐group sample sizes shown beneath each panel. Complete pairwise statistics are provided in Table S4.

Figure 4 shows a combined comparison across four glioma histomolecular subgroups defined by both molecular status and histological grade: 1p/19q‐codeleted oligodendrogliomas (Grades 2–3), non‐codeleted astrocytomas (Grade 2), non‐codeleted astrocytomas (Grades 3–4), and IDH‐wild‐type glioblastomas (Grade 4). Uncorrected APTw values increased non‐significantly from oligodendrogliomas through astrocytomas to IDH‐wild‐type gliomas. Following metrics correction, Grade 2 astrocytomas exhibited the lowest APTw‐FMC and APTw‐FMTC values: differences were significant between oligodendrogliomas and Grade 2 astrocytomas (adjusted *p* = 0.01 for APT‐FMC; *p* < 0.001 for APTw‐FMTC; |*δ*| = 0.62 and 0.78), and between Grade 2 astrocytomas and IDH‐wild‐type gliomas (adjusted *p* < 0.001 for both; |*δ*| = 1 for both), whereas oligodendrogliomas and higher‐grade astrocytomas did not differ significantly. NOEw‐based parameters showed no significant intergroup differences (all adjusted *p* > 0.77, |*δ*| < 0.28). Full statistics are provided in Table S4.

Several IDH‐mutant gliomas showed slightly negative APTw‐FMC and APTw‐FMTC values. This was more frequent in Grade 2 astrocytomas—[5/10] (50%) for APTw‐FMC and [10/10] (100%) for APTw‐FMTC—than in the other subgroups combined ([6/40] (15%) and [13/40] (32%), respectively).

## 4 Discussion

In this study, we aimed to elucidate the biophysical meaning of different APTw and NOEw contrasts and to evaluate their clinical utility for noninvasive glioma characterization at 3 T. Combining dual saturation powers (2 μT for APTw and 0.6 μT for NOEw) with an advanced preprocessing and a hierarchical correction pipeline, we systematically compared three CEST‐derived metrics (MTR_asym_, MTR_Rex_, and AREX) for their ability to predict IDH‐mutation and 1p/19q‐codeletion status in gliomas.

The Z‐spectra analysis reproduced the power‐dependent behavior described by Zhou et al. [29], with broader and deeper downfield APTw regime at high saturation (2 μT), reflecting stronger APT but also greater ssMT and DS (spillover contamination), and narrower upfield NOEw regime at low saturation (0.6 μT), with reduced DS and ssMT influence. This inverse relationship between APT and NOE contributions was consistent across patients, validating the use of dual‐power acquisitions to probe distinct exchange mechanisms. The hierarchical correction framework (MTR_asym_ → MTR_Rex_ → AREX) improved contrast specificity. MTR_Rex_ effectively suppressed spillover and ssMT background contributions, with the strongest effect observed in the APTw maps, while AREX further removed residual *T*_1_ dependence.

Quantitatively, all APT metrics differed significantly between IDH‐mutant and wild‐type gliomas, and both corrected metrics (APTw‐FMC, APTw‐FMTC) showed higher statistical significance and larger effect sizes than the uncorrected APTw metric. Mechanistically, the APTw signal scales with the concentration of mobile amide protons of peptides and proteins and with their base‐catalyzed exchange rate, which increases with intracellular pH. IDH‐wild‐type glioblastomas, characterized by high cellularity and proliferation, hypermetabolism, and fast protein synthesis, present a large mobile protein pool and correspondingly high amide signal, while IDH‐mutant gliomas, typically lower grade and less proliferative, present a smaller mobile protein pool and lower APTw signal. Moreover, IDH‐wild‐type gliomas have been shown to have slightly higher intracellular pH [30], which further contributes to increase the APTw signal. These observations support the interpretation that APTw contrast is elevated in more aggressive tumors.

Within the IDH‐mutant group, the difference between 1p/19q‐codeleted and non‐codeleted gliomas was strongly metric‐dependent, and notably the direction of the difference reversed after corrections. On uncorrected APTw metrics, astrocytomas showed higher signal than oligodendrogliomas, whereas on the fully corrected APTw‐FMTC the ordering inverted, with oligodendrogliomas showing the highest values (Figure 3). This reversal is the expected consequence of removing the dominant confounds: in astrocytomas, the apparently high uncorrected APTw may be partly driven by intratumoral fluid, microcystic, and edematous components. These compounds dilute the ssMT pool, alter DS/spillover and relaxation behavior, and thereby distort MTR_asym_ independently of true amide proton content. After correction for spillover, ssMT, and *T*_1_ contributions, the residual signal is expected to better reflect the mobile‐amide pool, which is higher in the compact, protein‐rich cellular architecture of oligodendrogliomas than in the fluid‐rich astrocytomas. These findings align with a previous report by Mancini et al. [10], suggesting that the apparent CEST contrast in IDH‐gliomas may be influenced by microcystic compartments with distinct localization—extracellular in astrocytomas and intracellular in oligodendrogliomas. In their study, IDH‐mutant, 1p/19q non‐codeleted astrocytomas frequently exhibited a *T*_2_w/FLAIR [31] mismatch together with an asymmetry‐ versus fluid‐suppressed mismatch in APTw maps, consistent with the coexistence of intra‐tumoral fluid‐like components that dilute the protein‐related signal. The behavior of APTw‐FMTC seems biologically complementary to the *T*_2_w/FLAIR mismatch sign, which helps identifying astrocytomas via the same fluid‐rich substrate but as a binary, qualitative sign; despite its utility as a marker for astrocytomas due to its very high specificity, the sensitivity of the T2‐FLAIR mismatch is however limited, as reported in a recent meta‐analysis [32].

The difference in APTw‐FMTC between astrocytomas and oligodendrogliomas did not survive FDR correction. To better understand this overlap, we then further stratified non‐codeleted astrocytomas in low‐grade and high‐grade subgroups (Figure 4). Uncorrected APTw increased with tumor aggressiveness, in line with previous studies focusing on the ability of APTw to assess tumor grading [4]. However, after spillover/ssMT and T1 corrections, Grade 2 astrocytomas showed the lowest APTw‐FMTC signal, while higher‐grade astrocytomas showed similar values as oligodendrogliomas. This could explain the weak differences between pooled astrocytomas and oligodendrogliomas, which was driven specifically by Grade 2 astrocytomas, and highlights the potential of APTw‐FMTC for identifying this subgroup.

Interestingly, several IDH‐mutant gliomas showed slightly negative APTw‐FMC and APTw‐FMTC values, indicating that these tumors exhibited a lower apparent signal than NAWM. As this effect was mainly observed in Grade 2 astrocytomas, it is consistent with the loosely packed, fluid‐rich cell architecture characteristic of these tumors, possibly combined with local intracellular pH variations [5, 8]. As the amide proton exchange rate is base‐catalyzed, a more acidic microenvironment reduces the exchange efficiency, resulting in lower APTw signal intensity. These results also support a semi‐quantitative rather than a purely visual analysis of APTw contrast.

In our study, the NOEw metrics did not differ between any molecular subgroups. This can be partially attributed to the distinct biophysical origin of the contrast where the upfield relayed NOE arises from dipolar cross‐relaxation of aliphatic protons of mobile macromolecules (membrane lipids and hydrophobic core of folded proteins), rather than from protein concentration or pH‐related changes in exchangeable amide protons [6]. Therefore, NOEw contrast likely reflects macromolecular membrane content and protein conformation more than the mobile‐protein concentration and acid–base changes that distinguish glioma molecular subtypes. Although the relayed NOE has shown tumor‐ and grade‐related changes in other studies—decreasing in glioma relative to normal brain and differing between low‐ and high‐grade gliomas, predominantly at 7 T [7, 17, 33]—it is intrinsically weaker and harder to isolate at 3 T, which may further limit its sensitivity here. In this sense, the absence of significant differences in NOEw metrics should not be interpreted as a lack of biological relevance, but rather as a limited sensitivity of this contrast for molecular stratification under clinical 3 T conditions.

This study has several limitations. The cohort size, although prospective, was single‐center and modest (*n* = 50), with uneven subgroup sizes, especially for the IDH‐wild‐type (*n* = 12) versus IDH‐mutants (*n* = 38) comparison. Analysis of histomolecular subgroups was exploratory, due to the limited sample sizes, especially for the high‐grade astrocytoma subgroup (*n* = 7). This limits the statistical power for intergroup comparisons. Moreover, the interpretations of our results, although in line with previous studies, remain speculative, as correlations of imaging metrics with histological measures of protein density, cellularity, or pH were not performed. Finally, larger, multicenter studies comparing the APTw‐FMTC metric with other imaging biomarkers for glioma subtyping are warranted to validate the added clinical utility of this metric.

## 5 Conclusion

This study demonstrated that corrected APTw CEST MRI metrics—accounting for spillover, semi‐solid MT effects, and *T*_1_ relaxation—enhance the performance of APTw imaging in differentiating glioma subgroups. Specifically, DS/ssMT‐corrected and *T*_1_‐compensated APTw metrics may enable more accurate differentiation between IDH‐mutant and IDH‐wild‐type gliomas. These corrected metrics also show potential in identifying the 1p/19q codeletion status, although further studies including larger cohorts are needed to validate our results. Overall, these findings support integrating corrected APTw imaging into routine glioma MRI protocols to improve the robustness and reliability of noninvasive tumor characterization.

## Author Contributions

**Capucine Cadin:** conceptualization, methodology, software, validation, formal analysis, investigation, data curation, visualization, writing – original draft. **Stefano Casagranda:** conceptualization, methodology, software, validation, formal analysis, investigation, data curation, visualization, writing – original draft. **François Xavier Lejeune:** supervision, software, formal analysis, data curation, writing – review and editing. **Marianne Golse:** formal analysis, validation, writing – review and editing. **Bertrand Mathon:** resources, investigation, project administration, writing – review and editing. **Ottavia Dipasquale:** formal analysis, software, validation, writing – review and editing. **Christos Papageorgakis:** formal analysis, software, validation, writing – review and editing. **Mauro Zucchelli:** formal analysis, software, validation, writing – review and editing. **Emmanuel Mandonnet:** resources, investigation, project administration, writing – review and editing. **Stéphane Lehéricy:** supervision, resources, investigation, project administration, writing – review and editing. **Franck Bielle:** resources, investigation, writing – review and editing. **Marc Sanson:** resources, investigation, project administration, writing – review and editing. **Patrick Liebig:** methodology, software, validation, writing – review and editing. **Moritz Zaiss:** methodology, software, validation, writing – review and editing. **Lucia Nichelli:** supervision, validation, project administration, resources, writing – review and editing. **Francesca Branzoli:** supervision, conceptualization, methodology, investigation, data curation, visualization, funding acquisition, writing – original draft.

## Funding

This work was supported by Agence Nationale de la Recherche (ANR‐20‐CE17‐0002‐01, ANR‐10‐IAIHU‐06, ANR‐11‐INBS‐0006, ANR‐11‐INBS‐0011).

## Conflicts of Interest

The authors declare no conflicts of interest related to this work.

S. Casagranda, O. Dipasquale, M. Zucchelli, and C. Papageorgakis are employees of Olea Medical. P. Liebig is an employee of Siemens Healthineers. L. Nichelli received speaker fees from Olea Medical.

## Supporting information


**Table S1:** Inter‐reader reproducibility of the APTw‐ and NOEw‐derived p90 metrics. Agreement between two independent readers (Reader 1 and Reader 2), who each delineated the tumor masks, assessed in all 50 patients. For each metric, the table reports the intraclass correlation coefficient (ICC (1, 2): two‐way random‐effects, single‐rater, absolute‐agreement model) with its 95% CI and qualitative agreement category, together with the Bland–Altman mean bias (Reader 1–Reader 2) and the 95% limits of agreement (LoA = bias ±1.96 SD). *Abbreviations*: ICC, intraclass correlation coefficient; CI, confidence interval; LoA, limits of agreement; SD, standard deviation; FMC, spillover/ssMT‐corrected; FMTC, spillover/ssMT‐ and *T*_*1*_‐corrected. Corresponding Bland–Altman plots are shown in Figure S1.
**Table S2:** Univariate association of APTw‐ and NOEw‐derived p90 metrics with IDH mutation status. For each metric, the table reports per‐group descriptive statistics (median [IQR]) in IDH‐wild‐type (*n* = 12) and IDH‐mutant (*n* = 38) gliomas, the between‐group comparison (two‐sided Mann–Whitney U test, with raw and Benjamini–Hochberg FDR‐adjusted *p*‐values across the six metrics), the Cliff's *δ* effect size with 95% CI, and discrimination performance (AUC with 95% CI and the Younden‐optimal threshold with sensitivity and specificity). *Abbreviations*: IQR, interquartile range; CI, confidence interval; BH‐FDR, Benjamini‐Hochberg false‐discovery rate; AUC, area under the ROC curve; Sens, sensitivity; Spec, specificity; FMC, fluid/ssMT‐corrected; FMTC, fluid/ssMT‐ and *T*_*1*_‐corrected. See footnotes for full statistical details.
**Table S3:** Univariate association of APTw‐ and NOEw‐derived p90 metrics with 1p/19q codeletion status (IDH‐mutant gliomas only). For each metric, the table reports per‐group descriptive statistics (median [IQR]) in non‐codeleted (*n* = 17) and codeleted (*n* = 21) IDH‐mutant gliomas, the between‐group comparison (two‐sided Mann–Whitney U test, with raw and Benjamini–Hochberg FDR‐adjusted *p*‐values across the six metrics), the Cliff's *δ* effect size with 95% CI, and discrimination performance (AUC with 95% CI and the Younden‐optimal threshold with sensitivity and specificity). *Abbreviations*: as in Table S2.
**Table S4:** Pairwise comparison of APTw‐ and NOEw‐derived p90 metrics across histomolecular glioma subgroups. For each metric, the table reports every pairwise comparison among the four subgroups (1p/19q ‐codeleted oligodendrogliomas grades 2–3, IDH‐mutant non‐codeleted astrocytomas grade 2, IDH‐mutant non‐codeleted astrocytomas grades 3–4, and IDH‐wild‐type glioblastomas). Each row gives the two groups compared, their sample sizes and descriptive statistics (median [IQR]), the two‐side Mann–Whitney U test (raw and Benjamini–Hochberg FDR‐adjusted *p*‐values, corrected across all pairwise comparisons within that metric), and the Cliff's *δ* effect size with 95% CI and magnitude. All pairwise comparisons are listed; only those significant after FDR correction (adjusted *p* < 0.05) are bracketed in Figure 4. *Abbreviations*: IQR, interquartile range; CI, confidence interval; BH‐FDR, Benjamini‐Hochberg false‐discovery rate; FMC, fluid/ssMT‐corrected; FMTC, fluid/ssMT‐ and *T*_*1*_‐corrected; ns, not significant. See footnotes for full statistical details.
**Figure S1:** Bland–Altman plots of inter‐reader agreement for the six APTw‐ and NOEw‐derived p90 metrics. For each metric, the difference between the two readers (Reader 1–Reader 2) is plotted against the mean of their two measurements (one point per patient, *n* = 50). The solid line indicates the mean bias and the dashed lines the 95% limits of agreement (bias ±1.96 SD). Limits of agreement are expressed in each metric's own units and are not directly comparable across metrics of different numeric scale: the wider limits for FMC/FMTC metrics reflect their larger value range, not poorer agreement, the scale‐free comparison is the ICC (all ≥ 90; Table S4). Corresponding ICCs and limits of agreement are reported in Table S1.

## Data Availability

The data that support the findings of this study are available upon request from the corresponding author. The data are not publicly available due to privacy or ethical restrictions.
