# Supplementary material for: Diagnostic Accuracy of APTw and NOEw MRI Metrics for IDH Mutation and 1p/19q Codeletion Prediction in Gliomas
Source: NMR Biomed. 2026 Aug 7;39(9):e70372. doi: 10.1002/nbm.70372 (PMC13449637; doi:10.1002/nbm.70372)
Supplement: Supplementary file 1 [file NBM-39-e70372-s001.docx]

**Supplementary material**

**Table S1: Inter-reader reproducibility of the APTw- and NOEw-derived p90 metrics.** Agreement between two independent readers (Reader 1 and Reader 2), who each delineated the tumor masks, assessed in all 50 patients. For each metric, the table reports the intraclass correlation coefficient (ICC(2,1): two-way random-effects, single-rater, absolute-agreement model) with its 95% CI and qualitative agreement category, together with the Bland-Altman mean bias (Reader 1 – Reader 2) and the 95% limits of agreement (LoA=bias±1.96 SD). *Abbreviations*: ICC, intraclass correlation coefficient; CI, confidence interval; LoA, limits of agreement; SD, standard deviation; FMC, spillover/ssMT-corrected; FMTC, spillover/ssMT- and *T_1_*-corrected. Corresponding Bland-Altman plots are shown in Figure S1.

| **Metric** | **n** | **ICC (95% CI)** | **Agreement** | **Mean bias** | **95% LoA** |
| --- | --- | --- | --- | --- | --- |
| APTw (p90) | 50 | 0.95 (0.91 to 0.97) | excellent | 0.054 | -0.394 to 0.503 |
| APTw-FMC (p90) | 50 | 0.94 (0.88 to 0.97) | excellent | 0.482 | -1.624 to 2.589 |
| APTw-FMTC (p90) | 50 | 0.96 (0.92 to 0.98) | excellent | 0.227 | -0.921 to 1.376 |
| NOEw (p90) | 50 | 0.91 (0.84 to 0.95) | excellent | 0.023 | -0.564 to 0.611 |
| NOEw-FMC (p90) | 50 | 0.90 (0.83 to 0.94) | good | -0.080 | -1.263 to 1.103 |
| NOEw-FMTC (p90) | 50 | 0.92 (0.86 to 0.95) | excellent | 0.042 | -0.681 to 0.765 |

ICC interpreted per Koo & Li (2016): <0.50 poor, 0.50-0.75 moderate, 0.75-0.90 good, >0.90 excellent.

LoA = limits of agreement (bias +/- 1.96 SD), Reader 1 - Reader 2.

**Figure S1: Bland-Altman plots of inter-reader agreement for the six APTw- and NOEw-derived p90 metrics.** For each metric, the difference between the two readers (Reader 1 – Reader 2) is plotted against the mean of their two measurements (one point per patient, n=50). The solid line indicates the mean bias and the dashed lines the 95% limits of agreement (bias ±1.96 SD). Limits of agreement are expressed in each metric’s own units and are not directly comparable across metrics of different numeric scale: the wider limits for FMC/FMTC metrics reflect their larger value range, not poorer agreement, the scale-free comparison is the ICC (all ≥ 90; Table S4). Corresponding ICCs and limits of agreement are reported in Table S1.

**Table S2: Univariate association of APTw- and NOEw-derived p90 metrics with IDH mutation status.** For each metric, the table reports per-group descriptive statistics (median [IQR]) in IDH-wild-type (n=12) and IDH-mutant (n=38) gliomas, the between-group comparison (two-sided Mann-Whitney U test, with raw and Benjamini-Hochberg FDR-adjusted p-values across the six metrics), the Cliff’s δ effect size with 95% CI, and discrimination performance (AUC with 95% CI and the Younden-optimal threshold with sensitivity and specificity). *Abbreviations*: IQR, interquartile range; CI, confidence interval; BH-FDR, Benjamini-Hochberg false-discovery rate; AUC, area under the ROC curve; Sens, sensitivity; Spec, specificity; FMC, fluid/ssMT-corrected; FMTC, fluid/ssMT- and *T_1_*-corrected. See footnotes for full statistical details.

| **Measure** | **n**  **(IDH wildtype/**  **IDH mutant)** | **IDH wildtype median [IQR]** | **IDH mutant median [IQR]** | **p (raw)** | **p (BH-FDR)** | **Signif.** | **Cliff's δ [95% CI]** | **d magnitude** | **AUC [95% CI]** | **Youden thr**  **(Sens, Spec)** |
| --- | --- | --- | --- | --- | --- | --- | --- | --- | --- | --- |
| APTw | 12/38 | 1.25 [0.71, 1.85] | 0.55 [0.26, 0.86] | 0.002 | 0.005 | ** | -0.570 [-0.799, -0.196] | large | 0.79 [0.63, 0.94] | 1.216 (0.89, 0.58) |
| APTw FMC | 12/38 | 4.44 [3.61, 5.53] | 0.83 [-0.24, 1.98] | <0.001 | <0.001 | *** | -0.886 [-0.979, -0.495] | large | 0.94 [0.87, 1.00] | 3.281 (0.95, 0.92) |
| APTw FMTC | 12/38 | 1.54 [1.30, 2.49] | -0.49 [-0.88, 0.26] | <0.001 | <0.001 | *** | -0.912 [-0.975, -0.715] | large | 0.96 [0.90, 1.00] | 0.958 (0.92, 1.00) |
| NOEw | 12/38 | 0.42 [0.09, 1.17] | 0.38 [0.02, 0.79] | 0.45 | 0.45 | ns | -0.149 [-0.491, 0.233] | small | 0.57 [0.38, 0.77] | - |
| NOEw FMC | 12/38 | 1.17 [0.13, 2.66] | 0.60 [-0.05, 1.48] | 0.25 | 0.37 | ns | -0.228 [-0.567, 0.177] | small | 0.61 [0.41, 0.82] | - |
| NOEw FMTC | 12/38 | 0.39 [0.20, 1.33] | 0.43 [-0.11, 0.93] | 0.40 | 0.45 | ns | -0.167 [-0.485, 0.191] | small | 0.42 [0.23, 0.61] | - |

Mann-Whitney U (Wilcoxon rank-sum) test, two-sided. n: patients with available data per group.

p adj: Benjamini-Hochberg FDR across the 6 metrics within this question; significance adjusted p < 0.05.

Cliff's delta with analytic 95% CI; positive delta indicates higher values in IDH mutant than IDH wild-type.

AUC (pROC, direction='auto' so orientation gives AUC >= 0.5; DeLong 95% CI). Youden-optimal threshold (sensitivity, specificity) shown only when the AUC 95% CI excludes 0.5.

Descriptives: median [interquartile range].

**Table S3: Univariate association of APTw- and NOEw-derived p90 metrics with 1p/19q codeletion status (IDH-mutant gliomas only).** For each metric, the table reports per-group descriptive statistics (median [IQR]) in non-codeleted (n=17) and codeleted (n=21) IDH-mutant gliomas, the between-group comparison (two-sided Mann-Whitney U test, with raw and Benjamini-Hochberg FDR-adjusted p-values across the six metrics), the Cliff’s δ effect size with 95% CI, and discrimination performance (AUC with 95% CI and the Younden-optimal threshold with sensitivity and specificity). *Abbreviations*: as in Table S2.

| **Measure** | **n**  **(Non-codeleted/**  **Codeleted)** | **Non-codeleted median [IQR]** | **Codeleted median [IQR]** | **p (raw)** | **p (BH-FDR)** | **Signif.** | **Cliff's δ [95% CI]** | **d magnitude** | **AUC [95% CI]** | **Youden thr**  **(Sens, Spec)** |
| --- | --- | --- | --- | --- | --- | --- | --- | --- | --- | --- |
| APTw | 17/21 | 0.61 [0.29, 1.10] | 0.52 [0.06, 0.77] | 0.28 | 0.48 | ns | -0.210 [-0.511, 0.137] | small | 0.61 [0.42, 0.79] | - |
| APTw FMC | 17/21 | 0.58 [-0.69, 1.00] | 1.40 [0.68, 2.10] | 0.06 | 0.17 | ns | 0.367 [0.016, 0.637] | medium | 0.68 [0.50, 0.86] | 0.678 (0.76, 0.59) |
| APTw FMTC | 17/21 | -0.83 [-1.41, -0.31] | 0.04 [-0.61, 0.50] | 0.01 | 0.06 | ns | 0.485 [0.142, 0.723] | large | 0.74 [0.57, 0.91] | -0.726 (0.86, 0.65) |
| NOEw | 17/21 | 0.55 [0.14, 0.75] | 0.22 [-0.14, 0.80] | 0.32 | 0.48 | ns | -0.193 [-0.514, 0.174] | small | 0.60 [0.41, 0.78] | - |
| NOEw FMC | 17/21 | 0.46 [0.16, 1.56] | 0.60 [-0.19, 1.33] | 0.93 | 0.93 | ns | -0.020 [-0.371, 0.336] | negligible | 0.49 [0.30, 0.68] | - |
| NOEw FMTC | 17/21 | 0.46 [-0.05, 0.93] | 0.28 [-0.24, 0.79] | 0.60 | 0.72 | ns | -0.104 [-0.453, 0.273] | negligible | 0.55 [0.36, 0.74] | - |

Mann-Whitney U (Wilcoxon rank-sum) test, two-sided. n: patients with available data per group.

p adj: Benjamini-Hochberg FDR across the 6 metrics within this question; significance adjusted p < 0.05.

Cliff's delta with analytic 95% CI; positive delta indicates higher values in Codeleted than Non-codeleted.

AUC (pROC, direction='auto' so orientation gives AUC >= 0.5; DeLong 95% CI). Youden-optimal threshold (sensitivity, specificity) shown only when the AUC 95% CI excludes 0.5.

Descriptives: median [interquartile range].

**Table S4: Pairwise comparison of APTw- and NOEw-derived p90 metrics across histomolecular glioma subgroups.** For each metric, the table reports every pairwise comparison among the four subgroups (1p/19q -codeleted oligodendrogliomas grades 2-3, IDH-mutant non-codeleted astrocytomas grade 2, IDH-mutant non-codeleted astrocytomas grades 3-4, and IDH-wild-type glioblastomas). Each row gives the two groups compared, their sample sizes and descriptive statistics (median [IQR]), the two-side Mann-Whitney U test (raw and Benjamini-Hochberg FDR-adjusted p-values, corrected across all pairwise comparisons within that metric), and the Cliff’s δ effect size with 95% CI and magnitude. All pairwise comparisons are listed; only those significant after FDR correction (adjusted p<0.05) are bracketed in Figure 4. *Abbreviations*: IQR, interquartile range; CI, confidence interval; BH-FDR, Benjamini-Hochberg false-discovery rate; FMC, fluid/ssMT-corrected; FMTC, fluid/ssMT- and *T_1_*-corrected; ns, not significant. See footnotes for full statistical details.

| **Metric** | **Comparison** | **n (g1/g2)** | **Median g1 [IQR]** | **Median g2 [IQR]** | **p (raw)** | **p adj (FDR)** | **Signif.** | **Cliff's δ [95% CI]** | **Magnitude** |
| --- | --- | --- | --- | --- | --- | --- | --- | --- | --- |
| APTw | Astro 2 vs Astro 3/4 | 10/7 | 0.52 [0.26, 1.01] | 0.81 [0.50, 1.29] | 0.23 | 0.34 | ns | -0.371 [-0.857, 0.200] | medium |
| APTw | Astro 2 vs IDH wild-type | 10/12 | 0.52 [0.26, 1.01] | 1.25 [0.71, 1.85] | 0.02 | 0.06 | ns | -0.583 [-0.917, -0.166] | large |
| APTw | Astro 3/4 vs IDH wild-type | 7/12 | 0.81 [0.50, 1.29] | 1.25 [0.71, 1.85] | 0.34 | 0.41 | ns | -0.286 [-0.810, 0.286] | small |
| APTw | Oligo 2/3 vs Astro 2 | 21/10 | 0.52 [0.06, 0.77] | 0.52 [0.26, 1.01] | 0.72 | 0.72 | ns | -0.086 [-0.552, 0.352] | negligible |
| APTw | Oligo 2/3 vs Astro 3/4 | 21/7 | 0.52 [0.06, 0.77] | 0.81 [0.50, 1.29] | 0.14 | 0.28 | ns | -0.388 [-0.769, 0.116] | medium |
| APTw | Oligo 2/3 vs IDH wild-type | 21/12 | 0.52 [0.06, 0.77] | 1.25 [0.71, 1.85] | 0.001 | 0.008 | ** | -0.659 [-0.913, -0.333] | large |
| NOEw | Astro 2 vs Astro 3/4 | 10/7 | 0.59 [0.23, 0.83] | 0.54 [0.09, 0.71] | 0.81 | 0.90 | ns | 0.086 [-0.457, 0.629] | negligible |
| NOEw | Astro 2 vs IDH wild-type | 10/12 | 0.59 [0.23, 0.83] | 0.42 [0.09, 1.17] | 0.87 | 0.90 | ns | 0.050 [-0.467, 0.550] | negligible |
| NOEw | Astro 3/4 vs IDH wild-type | 7/12 | 0.54 [0.09, 0.71] | 0.42 [0.09, 1.17] | 0.90 | 0.90 | ns | -0.048 [-0.595, 0.476] | negligible |
| NOEw | Oligo 2/3 vs Astro 2 | 21/10 | 0.22 [-0.14, 0.80] | 0.59 [0.23, 0.83] | 0.37 | 0.90 | ns | -0.210 [-0.638, 0.219] | small |
| NOEw | Oligo 2/3 vs Astro 3/4 | 21/7 | 0.22 [-0.14, 0.80] | 0.54 [0.09, 0.71] | 0.53 | 0.90 | ns | -0.170 [-0.592, 0.293] | small |
| NOEw | Oligo 2/3 vs IDH wild-type | 21/12 | 0.22 [-0.14, 0.80] | 0.42 [0.09, 1.17] | 0.20 | 0.90 | ns | -0.278 [-0.643, 0.103] | small |
| APTw FMC | Astro 2 vs Astro 3/4 | 10/7 | -0.05 [-1.63, 0.65] | 1.00 [0.42, 2.06] | 0.06 | 0.07 | ns | -0.571 [-0.943, -0.086] | large |
| APTw FMC | Astro 2 vs IDH wild-type | 10/12 | -0.05 [-1.63, 0.65] | 4.44 [3.61, 5.53] | <0.001 | <0.001 | *** | -0.983 [-1.000, -0.900] | large |
| APTw FMC | Astro 3/4 vs IDH wild-type | 7/12 | 1.00 [0.42, 2.06] | 4.44 [3.61, 5.53] | 0.010 | 0.01 | * | -0.714 [-1.000, -0.143] | large |
| APTw FMC | Oligo 2/3 vs Astro 2 | 21/10 | 1.40 [0.68, 2.10] | -0.05 [-1.63, 0.65] | 0.005 | 0.010 | ** | 0.619 [0.238, 0.886] | large |
| APTw FMC | Oligo 2/3 vs Astro 3/4 | 21/7 | 1.40 [0.68, 2.10] | 1.00 [0.42, 2.06] | >0.99 | >0.99 | ns | 0.007 [-0.551, 0.537] | negligible |
| APTw FMC | Oligo 2/3 vs IDH wild-type | 21/12 | 1.40 [0.68, 2.10] | 4.44 [3.61, 5.53] | <0.001 | <0.001 | *** | -0.897 [-1.000, -0.706] | large |
| NOEw FMC | Astro 2 vs Astro 3/4 | 10/7 | 0.35 [-0.01, 1.60] | 0.99 [0.27, 1.35] | 0.74 | 0.95 | ns | -0.114 [-0.657, 0.457] | negligible |
| NOEw FMC | Astro 2 vs IDH wild-type | 10/12 | 0.35 [-0.01, 1.60] | 1.17 [0.13, 2.66] | 0.46 | 0.95 | ns | -0.200 [-0.667, 0.300] | small |
| NOEw FMC | Astro 3/4 vs IDH wild-type | 7/12 | 0.99 [0.27, 1.35] | 1.17 [0.13, 2.66] | 0.59 | 0.95 | ns | -0.167 [-0.690, 0.381] | small |
| NOEw FMC | Oligo 2/3 vs Astro 2 | 21/10 | 0.60 [-0.19, 1.33] | 0.35 [-0.01, 1.60] | 0.95 | 0.95 | ns | 0.019 [-0.467, 0.467] | negligible |
| NOEw FMC | Oligo 2/3 vs Astro 3/4 | 21/7 | 0.60 [-0.19, 1.33] | 0.99 [0.27, 1.35] | 0.80 | 0.95 | ns | -0.075 [-0.551, 0.402] | negligible |
| NOEw FMC | Oligo 2/3 vs IDH wild-type | 21/12 | 0.60 [-0.19, 1.33] | 1.17 [0.13, 2.66] | 0.23 | 0.95 | ns | -0.262 [-0.659, 0.151] | small |
| APTw FMTC | Astro 2 vs Astro 3/4 | 10/7 | -1.02 [-2.74, -0.76] | 0.08 [-1.04, 0.49] | 0.07 | 0.08 | ns | -0.543 [-0.943, 0.029] | large |
| APTw FMTC | Astro 2 vs IDH wild-type | 10/12 | -1.02 [-2.74, -0.76] | 1.54 [1.30, 2.49] | <0.001 | <0.001 | *** | -1.000 [-1.000, -1.000] | large |
| APTw FMTC | Astro 3/4 vs IDH wild-type | 7/12 | 0.08 [-1.04, 0.49] | 1.54 [1.30, 2.49] | 0.007 | 0.01 | * | -0.738 [-1.000, -0.143] | large |
| APTw FMTC | Oligo 2/3 vs Astro 2 | 21/10 | 0.04 [-0.61, 0.50] | -1.02 [-2.74, -0.76] | <0.001 | <0.001 | *** | 0.781 [0.495, 0.952] | large |
| APTw FMTC | Oligo 2/3 vs Astro 3/4 | 21/7 | 0.04 [-0.61, 0.50] | 0.08 [-1.04, 0.49] | 0.84 | 0.84 | ns | 0.061 [-0.483, 0.633] | negligible |
| APTw FMTC | Oligo 2/3 vs IDH wild-type | 21/12 | 0.04 [-0.61, 0.50] | 1.54 [1.30, 2.49] | <0.001 | <0.001 | *** | -0.929 [-1.000, -0.786] | large |
| NOEw FMTC | Astro 2 vs Astro 3/4 | 10/7 | 0.46 [-0.21, 0.93] | 0.66 [0.04, 0.92] | 0.60 | 0.77 | ns | -0.171 [-0.714, 0.400] | small |
| NOEw FMTC | Astro 2 vs IDH wild-type | 10/12 | 0.46 [-0.21, 0.93] | 0.39 [0.20, 1.33] | 0.58 | 0.77 | ns | -0.150 [-0.633, 0.383] | small |
| NOEw FMTC | Astro 3/4 vs IDH wild-type | 7/12 | 0.66 [0.04, 0.92] | 0.39 [0.20, 1.33] | 0.77 | 0.77 | ns | -0.095 [-0.619, 0.477] | negligible |
| NOEw FMTC | Oligo 2/3 vs Astro 2 | 21/10 | 0.28 [-0.24, 0.79] | 0.46 [-0.21, 0.93] | 0.72 | 0.77 | ns | -0.086 [-0.524, 0.343] | negligible |
| NOEw FMTC | Oligo 2/3 vs Astro 3/4 | 21/7 | 0.28 [-0.24, 0.79] | 0.66 [0.04, 0.92] | 0.64 | 0.77 | ns | -0.129 [-0.592, 0.361] | negligible |
| NOEw FMTC | Oligo 2/3 vs IDH wild-type | 21/12 | 0.28 [-0.24, 0.79] | 0.39 [0.20, 1.33] | 0.36 | 0.77 | ns | -0.198 [-0.595, 0.214] | small |

Mann-Whitney U (Wilcoxon rank-sum) test, two-sided. n (g1/g2): number of patients per group.

p adj (FDR): Benjamini-Hochberg correction applied within each metric; significance threshold adjusted p < 0.05.

Effect size: Cliff's delta with 1000-resample bootstrap 95% CI; positive delta indicates group 1 > group 2 (negligible <0.147, small <0.33, medium <0.474, large >=0.474).

Descriptives reported as median [interquartile range].
